# Transdermal Delivery of Botulinum Toxin for the Management of Oily and Acne‐Prone Skin Using TDA Technology

**DOI:** 10.1111/jocd.70656

**Published:** 2026-02-06

**Authors:** Stephanie Schulz, Sophie Lamprecht

**Affiliations:** ^1^ Meddrop BioMedical Technologies GmbH Hamburg Germany; ^2^ Arc Clinic Barcelona Spain

## Abstract

**Background:**

Intradermal botulinum toxin injections have been shown to reduce sebaceous gland activity and improve skin texture in patients with oily and acne‐prone skin. However, needle‐based application is associated with discomfort and potential side effects.

**Objective:**

To evaluate the clinical efficacy and safety of transdermal delivery of botulinum toxin (150 kDa) using Transdermal Application (TDA) technology for the treatment of oily and acne‐prone skin.

**Methods:**

In this single‐center observational study, 19 participants (aged 20–50) underwent treatment with the DERMADROP MED TDA device according to the BIOBOTOX protocol. Objective assessments included Sebumeter measurements and 2D/3D skin imaging. Subjective evaluation was conducted using validated patient‐reported outcome measures (OSIS, OSSAS) at baseline, 2 weeks, and 4 weeks posttreatment.

**Results:**

A statistically significant reduction in sebum levels was observed, accompanied by notable improvements in self‐reported skin clarity and satisfaction. No adverse events were reported.

**Conclusion:**

Transdermal application of botulinum toxin using TDA‐based DERMADROP MED technology is a safe, well‐tolerated, and effective noninvasive treatment modality for reducing sebaceous activity and improving the appearance of oily and acne‐prone skin.

## 1 Introduction and Background

Intradermal microinjections of botulinum toxin type A have gained growing acceptance in aesthetic dermatology for improving skin quality in patients with seborrheic and acne‐prone skin. Clinical evidence supports their efficacy in reducing sebaceous gland activity, refining skin texture, and minimizing the appearance of pores. Standard protocols typically involve injecting diluted botulinum toxin into the superficial dermis, leading to measurable improvements in oil control and overall skin appearance [1, 2, 3, 4]. Despite its benefits, intradermal injection is associated with limitations such as procedure‐related discomfort, transient erythema, bruising, swelling, and, in rare cases, unintended effects on facial musculature due to diffusion of the toxin [5, 6]. These drawbacks have led to increased interest in noninvasive delivery alternatives that maintain efficacy while improving patient comfort and safety profiles.

The present case study investigates an alternative, noninvasive method for botulinum toxin delivery via the DERMADROP MED system, which employs a pressurized oxygen flow to facilitate needle‐free transdermal penetration of active compounds into deeper layers of the epidermis and superficial dermis. This investigation aims to assess the clinical efficacy and tolerability of transdermal botulinum toxin administration using this TDA‐based technology in patients exhibiting oily and acne‐prone skin phenotypes.

The DERMADROP MED TDA system is an approved noninvasive technology in aesthetic medicine and has been successfully used for the transdermal delivery of natural mesotherapy cocktails, peptides, and enzymes. Owing to its pain‐free application profile and the absence of posttreatment downtime, it has gained traction among patients seeking efficacious yet minimally invasive therapeutic approaches. Recent technological advances have enabled its adaptation for the dermal application of pharmacologically active agents such as botulinum toxin, thereby broadening its utility within the realm of noninvasive dermatologic interventions [7, 8].

It is hypothesized that TDA‐facilitated transdermal delivery of botulinum toxin constitutes a safe and effective alternative to intradermal injections, with the potential to attenuate sebaceous activity and improve overall skin appearance in individuals with oily and acne‐prone skin while minimizing the risks associated with invasive procedures.

## 2 Transdermal Application (TDA) Technology

**FIGURE 1:**
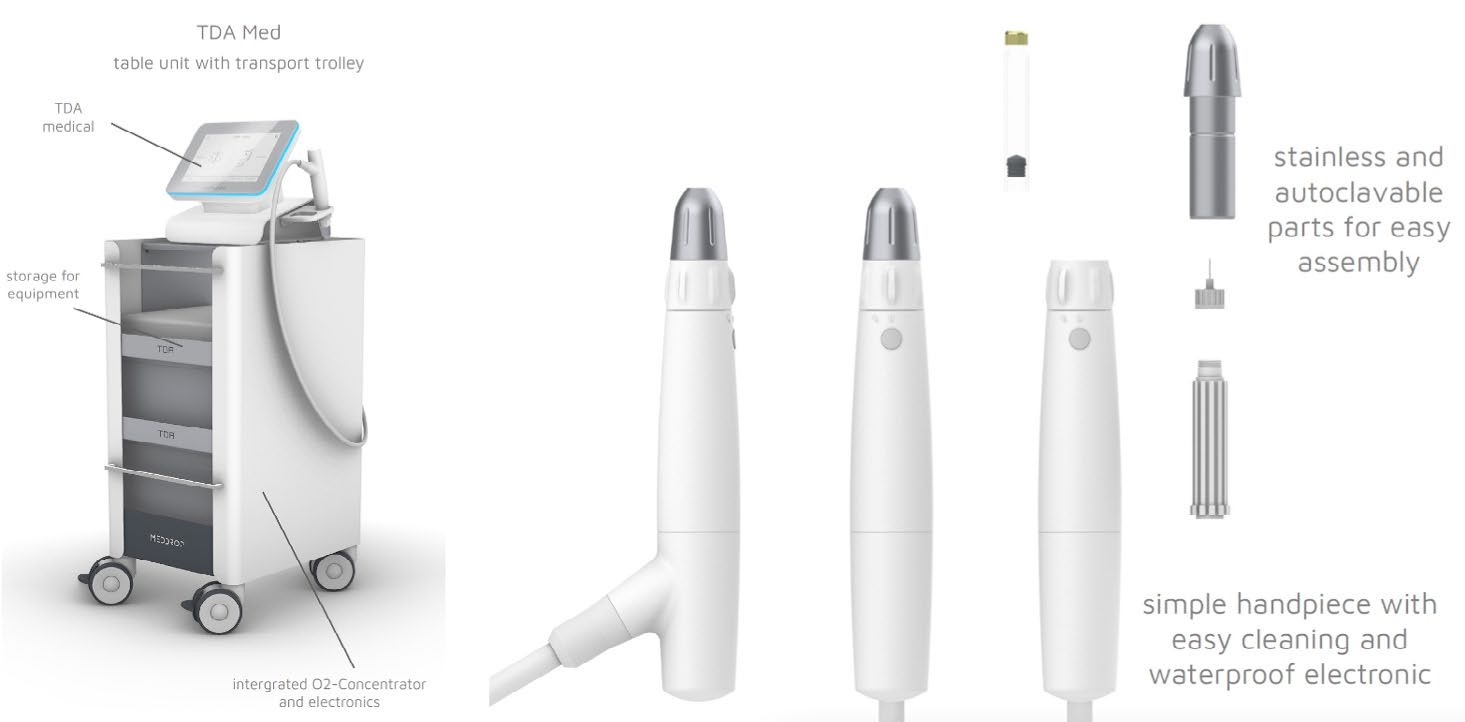
DERMADROP MED device and handpiece. Courtesy of Meddrop BioMedical Technologies GmbH, provided with permission (unpublished).

The DERMADROP MED system is based on MEDDROP BioMedical Technologies' proprietary TDA technology and is a method of noninvasive dermal drug delivery. The device (CE‐Class IIa) delivers active ingredients by means of a mechanically generated, oxygen‐assisted jet microapplication with a flow velocity of approximately 265 m/s, combined with a lipophilic–hydrophilic carrier matrix (Lipid Phase 3, LP3). This dual mechanism—(i) transient lamellar lipid disorganization induced by the carrier system and (ii) oxygen‐driven micro‐convective transport—facilitates trans‐epidermal passage without structural disruption of the skin barrier [9, 10]. As illustrated in Figure 1, the device comprises an oxygen generator, a computer‐controlled base unit, an applicator handpiece, and pre‐filled ampoules with TDA‐specific formulations. Earlier studies have demonstrated the feasibility and efficacy of this mechanism, confirming both the transient modulation of barrier lipids and the resulting improvement in transdermal delivery performance [11, 12, 13, 14, 15].

During application, oxygen extracted from ambient air is compressed, buffered, and fed into the applicator nozzle in a controlled manner. Within the nozzle, a focused high‐velocity microjet is generated, dispersing the carrier solution and the suspended active ingredients into ultra‐fine droplets and projecting them onto the skin with high‐precision. This process enables targeted deposition of active compounds into deeper epidermal layers without compromising the integrity of the stratum corneum. The applicator functions as a high‐precision nano‐disperser, with interchangeable cartridges that provide access to various therapeutic formulations. Upon activation, oxygen and carrier solution are mixed within the diffuser, after which the resulting dispersion is delivered through a microfine nozzle [10, 16].

The core component of the technology is the carrier substance (in this study the substance LP3 was used), a proprietary oil‐in‐water microemulsion. LP3 is a biphasic carrier matrix composed of phosphatidylcholine, polyglyceryl‐6 dioleate, and low‐molecular‐weight co‐solvents (propylene glycol, ethanol, aqua). It acts both as a transient barrier modulator and as a transport vehicle, forming oxygen‐enriched micro‐units within the applicator. These liposome‐like assemblies facilitate controlled penetration of hydrophilic and lipophilic substances through the stratum corneum. The LP3 vehicle contains phosphatidylcholine and short‐chain polyglycerides that transiently fluidize intercellular lipids, forming reversible diffusion channels [17]. This transient lipid reorganization enables micelles to permeate the stratum corneum lipid matrix [18]. Oxygen activation further enhances this permeability‐modulating effect and supports optimized transcutaneous uptake. Elevated oxygen partial pressure additionally drives micro‐convective transport and micro‐turbulent flow within follicular openings, which represent primary access sites to sebaceous gland structures [19].

In addition to increasing drug penetration, the carrier substance contributes to the formation of intradermal depots, supports the diffusion of molecular oxygen through the skin barrier and promotes prolonged bioavailability of the applied substances [20, 21]. The procedure is free from mechanical or thermal trauma and thus offers a safe and effective alternative to conventional transdermal application methods [22].

## 3 Methods

### 3.1 Study Design

This study was conducted in February/March 2024 at Regenera Clinic in Barcelona, Spain. The primary objective of the investigation was to evaluate the clinical efficacy of a needle‐free microapplication of botulinum toxin type A (Bocouture, 150 kDa, 25 IU) using the DERMADROP MED device, according to the standardized DERMADROP BIOBOTOX protocol. The study specifically focused on individuals with oily and acne‐prone skin, with the aim of reducing sebaceous gland activity and improving overall skin quality. A total of 19 subjects (15 female, 4 male) were enrolled, all of whom met the defined inclusion criteria designed to ensure methodological consistency and the validity of outcomes. All participants provided written informed consent prior to study inclusion. This included consent to participate in the investigation as well as to the anonymized processing and scientific analysis of the collected data and, where applicable, any recorded photographic material.

#### 3.1.1 Inclusion Criteria

Participants were eligible for inclusion if they fulfilled the following criteria:

- Age between 20 and 50 years
- No prior facial interventions (e.g., microneedling, ablative or non‐ablative laser therapy, chemical peels, dermabrasion, mesotherapy, platelet‐rich plasma treatment) within 60 days prior to study initiation
- No use of topical agents known to modulate sebaceous activity (e.g., retinoids) within 30 days before treatment
- Absence of artificial tanning
- No botulinum toxin treatment in the target area within the 30 days preceding study onset

Any deviation from the abovementioned criteria resulted in exclusion from the study population.

#### 3.1.2 Exclusion Criteria

In addition to the failure to meet inclusion criteria, subjects were excluded if any standard contraindications to botulinum toxin were present. These included known hypersensitivity reactions to botulinum toxin or its excipients, active dermal infections or inflammatory dermatoses in the treatment area, a history of autoimmune or neuromuscular disorders, or current pregnancy or lactation.

### 3.2 Indications

The clinical indications addressed in this study comprise dermatologic conditions characterized by excessive sebum production and associated textural irregularities. Specifically, subjects presented with oily skin, enlarged pilosebaceous units (visible as large pores), impurities, and a predisposition to acneiform eruptions.

### 3.3 Outcomes

The primary outcome measure was defined as the overall improvement in skin quality in individuals with seborrheic skin. This was operationalized through a clinically observable and subjectively reported reduction in sebum production, reflecting the anticipated therapeutic effect of the botulinum toxin–based intervention.

### 3.4 Treatment Procedure

In order to ensure both therapeutic efficacy and procedural safety in the context of noninvasive transdermal botulinum toxin delivery using the DERMADROP MED system, a rigorously standardized protocol was implemented. The protocol consisted of the following sequential steps:

1. Facial cleansing prior to treatment: Subjects were instructed to remove any facial cosmetics on the evening prior to treatment. On the day of the procedure, they were advised to cleanse the face with a warm, damp towel approximately 3 h before the appointment, to refrain from applying any skincare products thereafter, to avoid touching the face, engaging in physical exercise, or exposing the skin to direct sunlight.
2. Cutaneous acclimatization: Upon arrival, subjects were allowed a 30‐min acclimatization period to standardize skin temperature and baseline conditions prior to imaging and treatment.
3. Baseline data collection: Prior to the intervention, high‐resolution 2D and 3D facial images were obtained. Subjects completed standardized questionnaires, and sebum production was quantified using a Sebumeter.
4. Antiseptic skin preparation: The facial area was disinfected with a chlorhexidine‐based antiseptic solution to minimize the risk of contamination.
5. Mechanical preconditioning: A standardized microdermabrasion procedure was performed to remove excess sebum and superficial keratinized cells that could interfere with uniform application. This step ensured standardized surface conditions, providing comparable baseline roughness and absorption characteristics across participants. This gentle exfoliation method enhances cutaneous microcirculation and optimally prepares the skin for transdermal substance delivery.
6. Formulation preparation: The botulinum toxin solution was prepared according to the DERMADROP BIOBTX 1 protocol by reconstituting 50 IU of Bocouture (150 kDa) in 1.3 mL of a 10% Argireline‐containing solution. The solution was gently homogenized and 0.65 mL (corresponding to 25 IU per subject) was transferred into a sterile, single‐use DERMADROP cartridge. Argireline is an acetylated hexapeptide incorporated as a stabilizing and surface‐active component for toxin reconstitution. At the applied concentration (10%), it does not alter the fundamental transport characteristics of the LP3 carrier but primarily facilitates uniform dispersion of the reconstituted botulinum solution and provides minor surface‐relaxing effects.
7. TDA‐based treatment application: The treatment was administered using the DERMADROP MED device under defined technical parameters, enabling high‐velocity, needle‐free transdermal penetration of the active formulation.
8. Posttreatment care: Following the procedure, a Cover‐Hyal‐Mask was applied. This bioactive formulation supports enhanced percutaneous hydration, promotes epidermal regeneration, and facilitates rapid recovery. Its use served exclusively to improve participant comfort and skin hydration; it has no pharmacological relevance for toxin delivery. The cooling effect reduces transient dryness or tension in sensitive subjects and aligns with standard practice in noninvasive aesthetic procedures.

To prolong the exposure time of the active compounds and to protect the skin post‐intervention, participants were instructed to adhere to the following posttreatment instructions:

- Same day of your treatment:
  ○ Refrain from touching the face for approximately 4 h
  ○ Avoid applying cosmetic products, including make‐up
  ○ Abstain from physical exertion or sweating
  ○ Do not wash the face until the next morning
- For the 2 weeks following treatment:
  ○ Avoid direct sun exposure for at least 48 h
  ○ Refrain from sauna use, steam baths, or immersion in hot water during this period
  ○ Observe skin changes and complete follow‐up questionnaire either digitally (via WhatsApp) or as a printed copy during the follow‐up appointment

The transdermal botulinum toxin application via the DERMADROP MED system was consistently reported by participants as highly tolerable and subjectively pleasant. In contrast to conventional injection‐based aesthetic procedures, the TDA‐based intervention was not associated with common adverse effects such as erythema, edema, or pain and thus required no post‐procedural downtime. All subjects reported a positive sensory experience throughout the treatment process.

### 3.5 Technical Configuration

The intervention was performed according to a standardized 12‐min protocol, employing region‐specific settings for flow rate, dosage, and frequency to accommodate the anatomical and functional characteristics of distinct facial areas, including the forehead, cheeks, periorbital region (crow's feet), infraorbital area, glabellar complex (frown lines), and nasal dorsum. The application angle of the DERMADROP MED handpiece was maintained at 90°, with a working distance of approximately 1.5–2 cm from the skin surface to ensure homogeneous and targeted distribution of the botulinum toxin–saline–LP3 formulation.

- Average flow rate: 55 μL/min
- Total amount: 650 μL

### 3.6 Data Collection

Data acquisition was performed at baseline (prior to treatment) and 2 weeks post‐intervention. At both time points, standardized imaging and biophysical measurements were conducted, and two validated patient‐reported outcome measures were administered. A final questionnaire assessment was repeated 4 weeks posttreatment to capture longer‐term subjective outcomes.

#### 3.6.1 3D Images

Three‐dimensional surface imaging was conducted using the *QuantifiCare Mini* system. The device captures multiple images from varying angles, which are algorithmically merged into high‐resolution 3D renderings using QuantifiCare software. This allows for precise visual comparison of defined facial regions across time points. All imaging procedures adhered to the manufacturer's specifications regarding lighting, distance, and calibration.

#### 3.6.2 2D Images

Supplementary 2D photographic documentation was performed using a *Sony Alpha NEX‐5* digital camera equipped with a 50 mm fixed focal length lens. Images were acquired in a controlled photographic setup including a standardized blue background, dual external flash units, and a tripod‐mounted camera. Each subject was photographed in five views: frontal, 45° oblique right and left, and true profile (90°) right and left. These images served for blinded expert evaluation using a predefined clinical rating scale.

#### 3.6.3 Oily Skin Impact Scale (OSIS)

The OSIS is a validated instrument designed to assess the psychosocial impact of oily facial skin. It consists of six items grouped into two subdomains: *Annoyance* (three items) and *Self‐Image* (three items). Responses are recorded on a 10‐point Likert scale ranging from “not at all” (0) to “extremely” (10), providing a composite measure of emotional burden associated with seborrheic skin [23, 24].

#### 3.6.4 Oily Skin Self‐Assessment Scale (OSSAS)

The OSSAS is a self‐administered questionnaire that evaluates subjective perception of facial oiliness. It includes 14 items across four assessment modalities: *visual* (3 items), *tactile* (4 items), *sensory* (5 items), and *blotting‐based perception* (1 item), along with one overall severity rating. All items are rated on a 10‐point scale (“not at all” to “extremely”). The scale offers a comprehensive self‐assessment of oily skin symptoms and is validated for use in dermatological studies [23, 24].

#### 3.6.5 Sebumeter

Quantitative assessment of cutaneous sebum levels was conducted using *Sebumeter SM815* (Courage + Khazaka electronic GmbH, Cologne, Germany), a validated instrument for direct photometric measurement of surface lipids. Sebum output was expressed in mg/cm^2^. For each participant, the two facial regions subjectively identified as the most seborrheic were selected for measurement. Following the manufacturer's standardized operating procedure, three consecutive readings were obtained per measurement site, and the arithmetic mean was calculated. A tolerance range of 30 (as specified by the manufacturer) was applied to ensure measurement reliability. All procedures were carried out under controlled environmental conditions to minimize variability due to temperature, humidity, or skin preconditioning effects.

The reference values provided by the manufacturer are summarized in Table 1.

**TABLE 1:** Sebumeter reference values [25].

	Forehead, t‐zone	Cheek
Dry	< 100	< 70
Normal	100–180	70–150
Oily	> 180	> 150

## 4 Results

Normal distribution of all continuous variables was confirmed using the Shapiro–Wilk test; therefore, paired samples *t*‐tests were applied for inferential statistical analysis.

Transdermal microapplication of botulinum toxin via the TDA‐based DERMADROP MED system resulted in statistically and clinically relevant improvements in overall skin quality, as evidenced by both objective biometric measurements and subjective patient‐reported outcomes. The combined analysis of Sebumeter data, photographic documentation, and standardized questionnaires supports the efficacy of this noninvasive delivery method in reducing sebaceous gland activity and improving skin clarity.

### 4.1 Sebumeter

Quantitative sebum measurements revealed a mean reduction in surface oiliness across all assessed facial regions. The most pronounced effect was observed in the forehead, where mean Sebumeter values decreased from 166.75 mg/cm^2^ to 100.19 mg/cm^2^, representing a 39.92% reduction (*t* = 4.32, df = 15, *p* < 0.001).

Further decreases were noted in the following regions:

- Cheeks: from 107.40 to 70.80 mg/cm^2^ (reduction of 34.08%)
- Chin: from 116.33 to 78.67 mg/cm^2^ (32.38% reduction)
- Nasal alae: from 146.88 to 108.83 mg/cm^2^ (25.91% reduction)

While all areas showed numerical improvements, statistically significant reductions were only achieved in the forehead region. The reductions in cheek, chin, and nasal areas did not reach statistical significance.

### 4.2 Questionnaire Analysis

Data from validated patient‐reported outcome measures were analyzed using *JASP* software (Version 0.18.3; jasp‐stats.org).

#### 4.2.1 Oily Skin Impact Scale (OSIS)

Significant reductions were observed across all six items of the OSIS. In the *Annoyance* domain, mean scores decreased from 5.50 to 2.30 (a 58% reduction), while the *Self‐Image* domain showed a decrease from 4.90 to 2.23 (54% reduction).

The greatest absolute improvements were observed in the following single‐item measures:

- Frustration (“Over the past week, how much did the oiliness of your face make you feel frustrated?”): mean reduction of 3.32 points (*t* = 5.26, df = 18, *p* < 0.001)
- Annoyance (“Over the past week, how much did the oiliness of your face make you feel annoyed?”): mean reduction of 3.58 points (*t* = 5.26, df = 18, *p* < 0.001)

**FIGURE 2:**
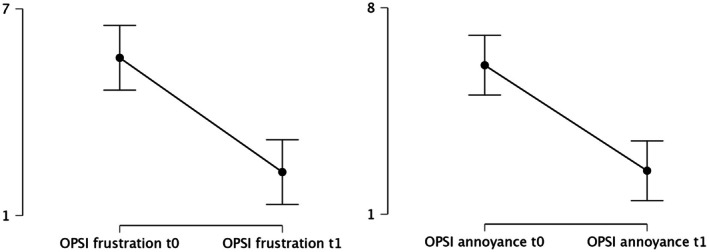
Scatter plots of the OSIS categories frustration and annoyance.

Both items exhibited a relative improvement of approximately 59%, as depicted in Figure 2, thereby underscoring the emotional and psychosocial benefit of the intervention.

#### 4.2.2 Oily Skin Self‐Assessment Scale (OSSAS)

Analysis of the OSSAS revealed statistically significant improvements in perceived skin oiliness across all assessed domains: *visual*, *sensory*, and *tactile* evaluation.

- In the visual domain, mean scores decreased from 5.30 to 2.80, corresponding to a 48% reduction
- In the sensory domain, scores declined from 5.10 to 2.20 (57% reduction)
- In the tactile domain, mean values fell from 5.90 to 3.00 (49% reduction)

Evaluation of the single‐item measure involving blotting paper application to the forehead (“How oily is the blotting paper?”) demonstrated a mean improvement of 3.44 points (*t* = 6.65, df = 18, *p* < 0.001).

**FIGURE 3:**
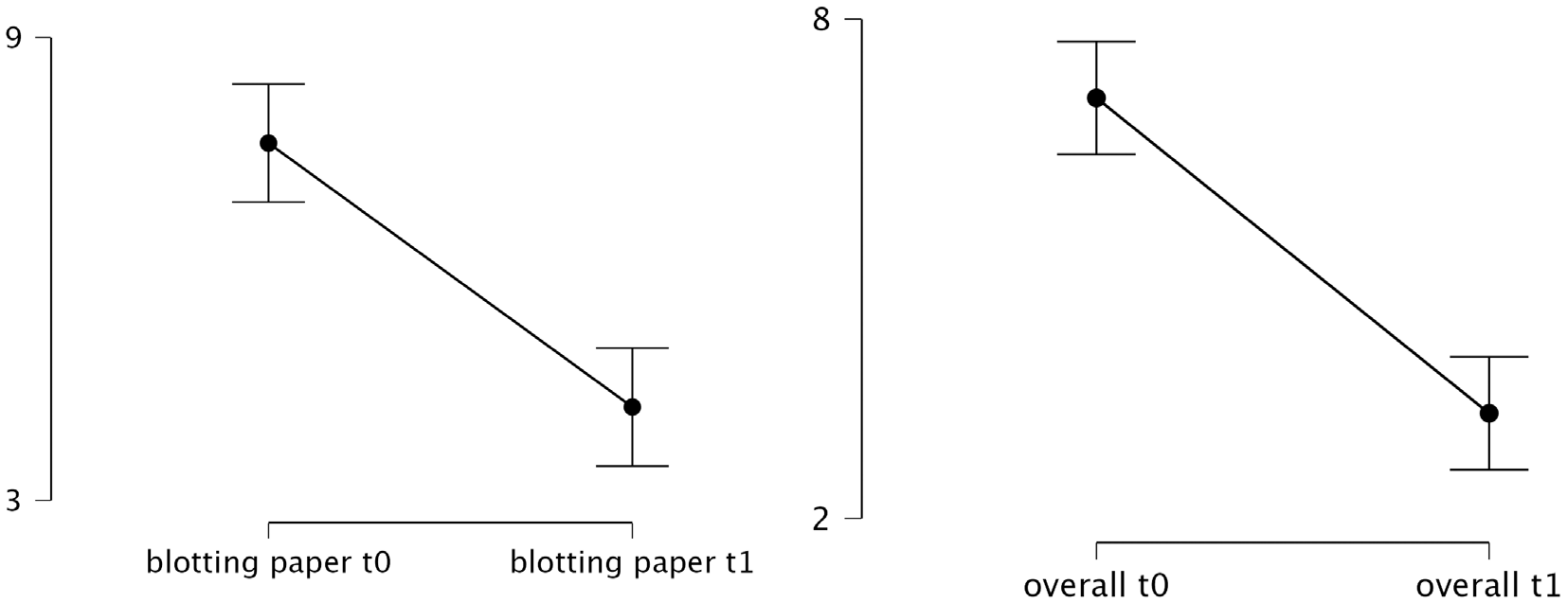
Scatter plots of the OSSAS categories blotting paper and overall impression.

Moreover, the participants' overall self‐assessment of facial oiliness showed a mean improvement of 3.79 points (*t* = 8.31, df = 18, *p* < 0.001), highlighting a substantial subjective benefit following the intervention. Figure 3 displays the scatter plots for the blotting paper measurements and the overall self‐assessment.

### 4.3 Treatment Tolerability and Satisfaction

Participants consistently reported perceived improvements in skin texture and reductions in seborrheic activity. Individuals exhibiting visible clinical changes further described an enhancement in skin luminosity, increased evenness of skin tone, and a general improvement in skin quality, which collectively resulted in a decreased reliance on cosmetic makeup. In addition to the predefined outcome measures, participants also spontaneously reported secondary benefits not initially targeted by the study. These included improvements in hyperpigmentation (reduction of pigmentation spots) and attenuation of periorbital dark circles, which were documented accordingly for exploratory analysis. The transdermal application of botulinum toxin using the DERMADROP MED system was uniformly well tolerated across the study cohort. Unlike conventional facial aesthetic interventions, the TDA‐based method was not associated with procedure‐related adverse effects such as erythema, edema, or pain and did not necessitate posttreatment downtime. All participants rated the treatment experience positively and expressed high overall satisfaction with the procedural comfort and outcomes.

### 4.4 Duration of the Effect

At the four‐week follow‐up, questionnaire data were available for eight participants due to logistical limitations in participant tracking.

Analysis of OSSAS scores indicated further mean improvements in six out of fourteen evaluated items when compared to the 2‐week posttreatment assessment. In the remaining domains, scores either stabilized or exhibited minor declines; however, all values remained significantly improved relative to baseline.

Similarly, OSIS scores demonstrated continued enhancement across all assessed items at the 4‐week time point compared to the 2‐week evaluation. These findings collectively suggest that the therapeutic benefits of transdermal botulinum toxin application via the DERMADROP MED system are sustained for at least 4 weeks following a single treatment session.

### 4.5 Case Presentation

To provide a clearer understanding of the observed changes and treatment effects, we have selected individual cases for detailed illustration.

#### 4.5.1 Case 1

**FIGURE 4:**
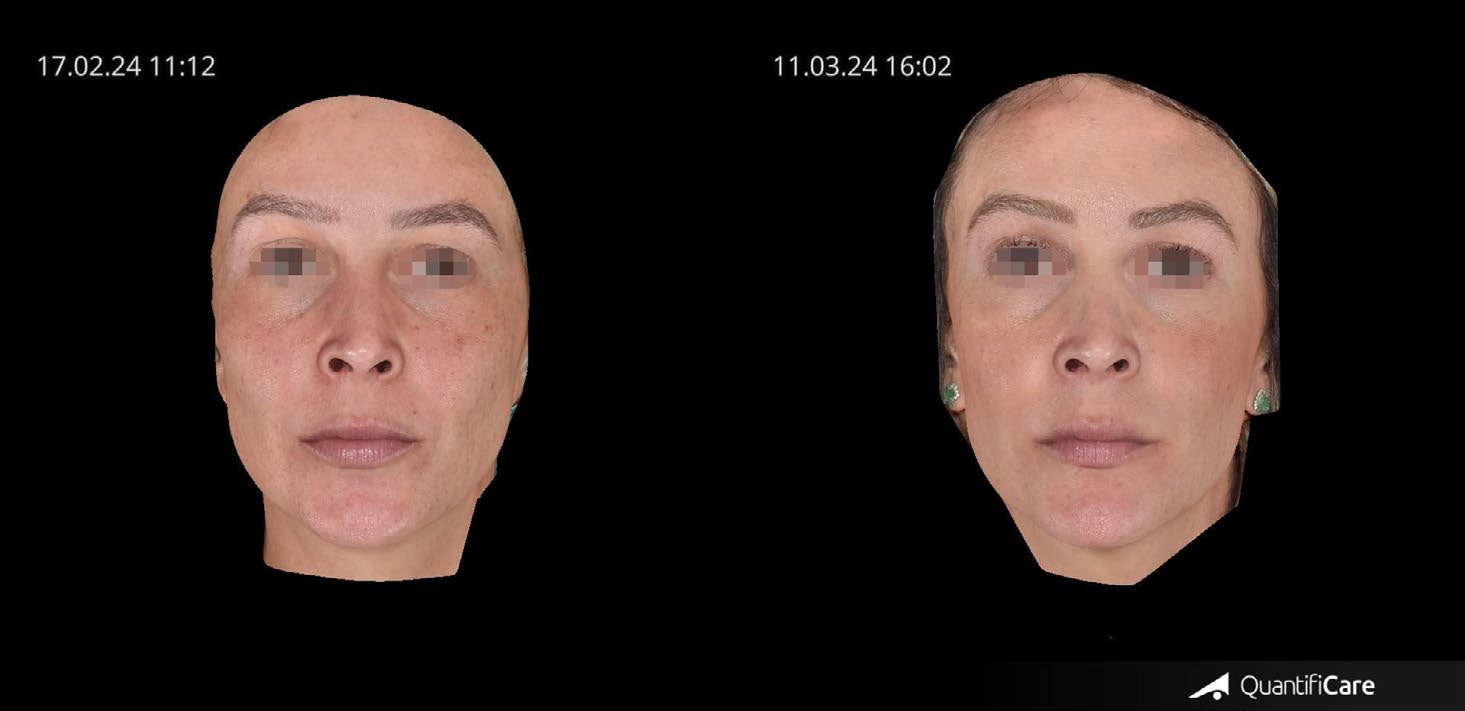
Pre and posttreatment images of a participant treated with transdermal Botulinum Toxin using TDA technology, showing a clearer and more even facial skin appearance.

As shown in Figure 4, this participant demonstrated a marked improvement in the overall appearance of facial skin, characterized by a reduction in pore size and a more even, refined, and clearer complexion.

#### 4.5.2 Case 2

**FIGURE 5:**
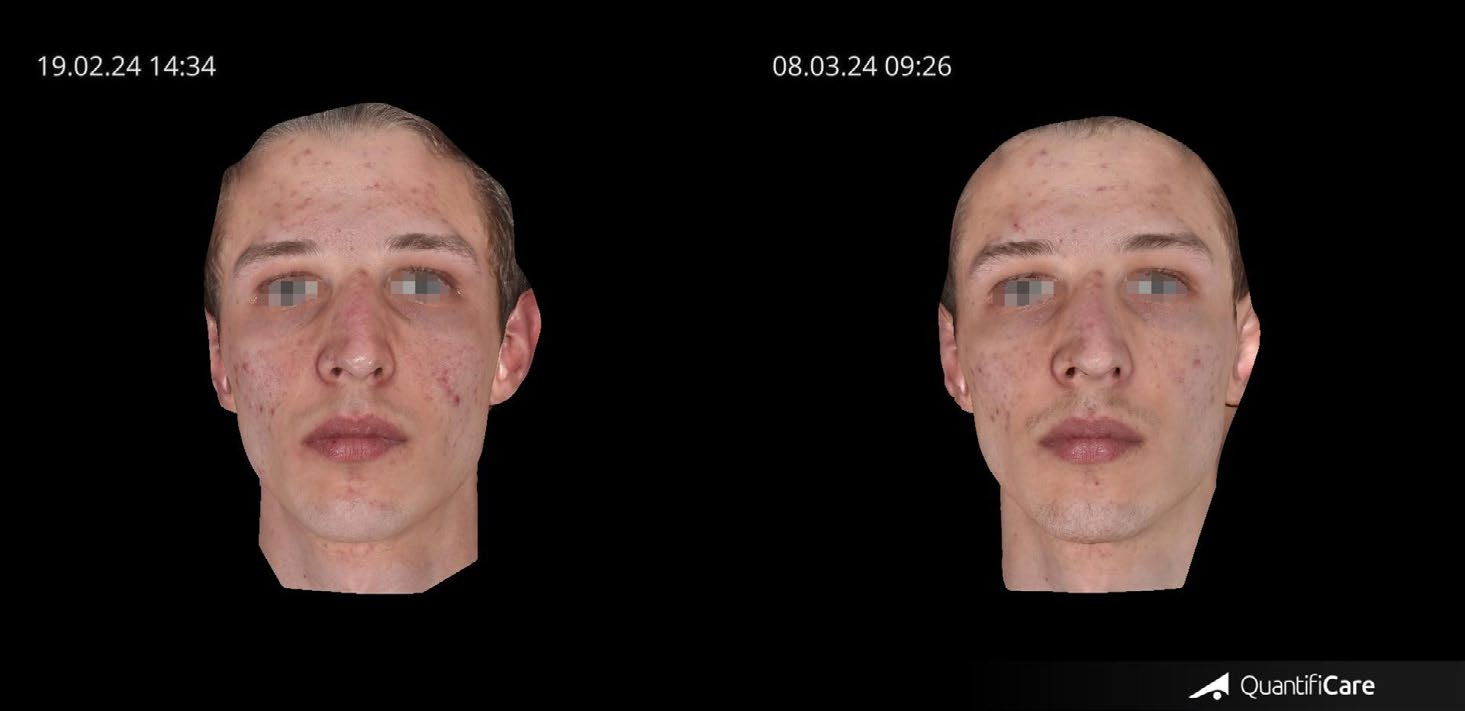
Pre and posttreatment images of a participant treated with transdermal Botulinum Toxin using TDA technology, showing a decrease in acne activity.

Figure 5 shows that this subject exhibited a notable enhancement in overall skin condition, including a significant decrease in acne activity and visibly reduced redness and inflammation.

#### 4.5.3 Case 3

**FIGURE 6:**
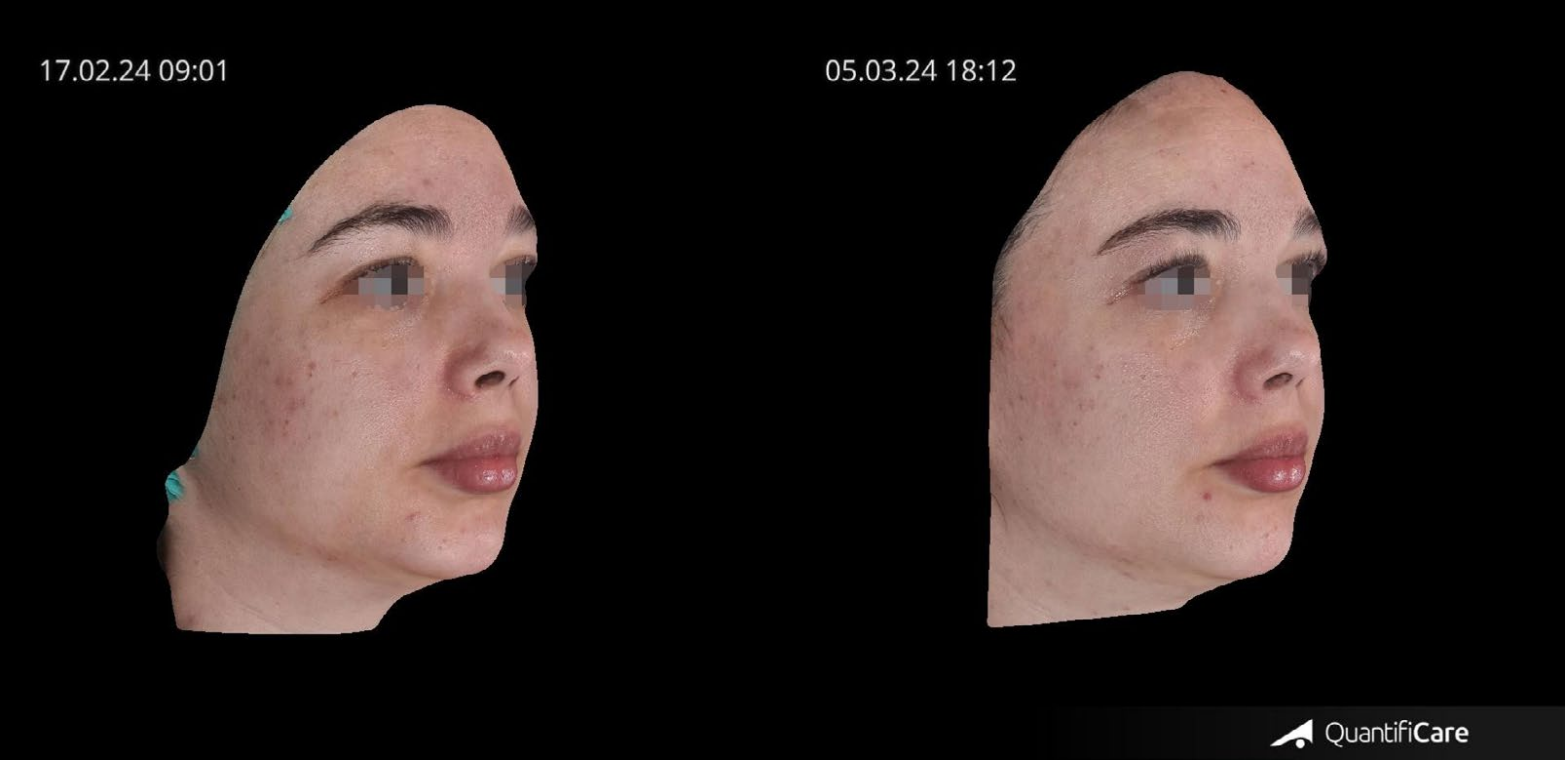
Pre and posttreatment images of a participant treated with transdermal Botulinum Toxin using TDA technology, showing a clearer and fresher overall skin appearance.

This participant showed clearly enhanced skin quality, particularly in texture and tone, as demonstrated in Figure 6. Previously oily skin, especially in the T‐zone, appeared noticeably less greasy following the treatment, and the participant subsequently reported a reduced need for the at‐home skincare products that were part of their usual routine.

## 5 Discussion and Expert Opinion

This study aimed to evaluate the efficacy and safety of transdermal botulinum toxin application using the TDA‐based DERMADROP MED technology for enhancing skin quality in individuals with oily and acne‐prone skin. The findings demonstrate that this noninvasive, needle‐free delivery method yields significant improvements in both objective and subjective indicators of seborrhea, while also offering excellent tolerability and patient satisfaction.

In recent years, intradermal microinjections of botulinum toxin have gained attention as an off‐label method to reduce facial sebum production. The mechanism is thought to involve inhibition of acetylcholine‐mediated stimulation of the sebaceous glands, resulting in downregulation of sebum output [2, 3]. While effective, traditional injection‐based approaches are associated with discomfort, posttreatment erythema, bruising, and the risk of unintended muscle paralysis when not precisely administered [6, 26]. Our findings indicate that transdermal delivery, specifically via the TDA system, can offer comparable benefits without these disadvantages.

Significant improvements were documented through objective Sebumeter measurements as well as validated patient‐reported outcome measures (OSIS and OSSAS). Participants reported a reduction in sebaceous activity, improved skin texture, and improved radiance. Statistically, reductions in emotional burden related to oily skin, particularly frustration and annoyance, were notable, with mean score improvements exceeding 3 points on a 10‐point scale. These findings are in line with previous literature indicating that oily skin has not only physiological but also considerable psychosocial implications [26, 27].

The OSSAS results further corroborated a reduction in perceived facial oiliness, with the blotting paper test and overall self‐assessment scores improved significantly. This reflects the participants' subjective experience of oil control, as reported in prior botulinum toxin studies, but achieved here through a needle‐free approach. Importantly, our approach also preserved epidermal integrity and high treatment tolerability throughout the intervention.

In terms of safety, no severe or lasting adverse effects were reported. Mild posttreatment dryness occurred in participants with predisposing factors such as sensitive or atopic skin but resolved within 3 days. Importantly, no flare‐ups of underlying skin conditions such as acne, rosacea, or dermatitis were observed. These findings reinforce the favorable safety profile of the TDA transdermal method and support its applicability across a broader dermatological spectrum. This may represent a clinical advantage over more invasive methods, especially for patients contraindicated for injectables or seeking minimal downtime.

An unexpected but clinically relevant observation was the improvement in pigmentation irregularities and under‐eye darkness in some participants. While these effects were not primary endpoints, they warrant further exploration. The mechanism could relate to enhanced microcirculation or improved delivery of oxygen due to the DERMADROP TDA technology, which is known to support dermal matrix health and even pigmentation [7, 17].

The study has some limitations. First, the sample size was modest, and the follow‐up period was relatively short. Long‐term durability of the results beyond 4 weeks remains to be studied. Second, while the use of imaging systems and validated scales adds objectivity, the study would benefit from blinded expert panel evaluation and possibly histological analysis in future trials.

Despite these limitations, this study provides clinically relevant evidence supporting a safe and effective noninvasive alternative for managing oily skin through botulinum toxin. Given the growing patient demand for needle‐free, low‐downtime aesthetic treatments, transdermal application of botulinum toxin via systems such as DERMADROP MED may offer a highly attractive solution.

Future research should focus on larger randomized controlled trials, explore optimal dosing regimens and treatment intervals, and further investigate potential benefits for related conditions such as enlarged pores, mild acne, or early photoaging. Additionally, mechanistic studies exploring the exact pathways through which botulinum toxin exerts its effects when applied transdermally will be critical to understanding and optimizing this novel delivery method.

## Author Contributions

Stephanie Schulz contributed to the development of the methodology, performed data analysis, participated in data acquisition, and was involved in writing and editing the manuscript. Dr. Sophie Lamprecht screened patients for eligibility, conducted the treatments, contributed to data acquisition, and participated in writing and revising the manuscript. Provided overall supervision of the clinical aspects of the study.

## Funding

This work was supported by Meddrop BioMedical Technologies GmbH.

## Ethics Statement

All participants provided written informed consent prior to inclusion. As a noninvasive, observational case study without the use of experimental pharmaceuticals, formal IRB approval was not required under Spanish research guidelines.

## Consent

All participants provided written informed consent prior to enrollment, including consent for the use and publication of their clinical photographs for scientific and academic purposes.

## Conflicts of Interest

The study was conducted under the auspices of Meddrop BioMedical Technologies GmbH, which also served as the study sponsor. Stephanie Schulz is an employee of the sponsoring company. Dr. Sophie Lamprecht received funding for the study coordination. No other conflicts of interest are reported.

## Data Availability

The data that support the findings of this study are available from the corresponding author upon reasonable request.
